# Shades of evidence: a review of skin color reporting in melanoma-related randomized controlled trials

**DOI:** 10.1007/s00403-025-03793-z

**Published:** 2025-01-16

**Authors:** Deven P. Curtis, Natasha L. Salmen, Anthony N. Baumann, Justine F. Busby, Robert T. Brodell

**Affiliations:** 1 College of Medicine, Northeast Ohio Medical University, 4209 OH-44, OH 44272 Rootstown, USA; 2 University of Mississippi Medical Center, Mississippi and JV “Sonny” Montgomery Veterans Hospital, Jackson, MS USA

**Keywords:** Melanoma, Cutaneous malignant melanoma, Skin of Color (SOC), Fitzpatrick Scale

## Abstract

**Objectives:**

To examine the rate of skin color reporting in randomized controlled trials (RCTs) involving melanoma in the top ten highest dermatology journals by impact factor over the past four decades.

**Methods:**

A systematic review of RCTs involving melanoma within the top ten dermatology journals, as determined by impact factor, was conducted from inception to July 10th, 2023. Studies were included if they reviewed the diagnosis and/or treatment of melanoma, were RCTs, directly involved patients and were written in English. Studies were characterized as positive for reporting skin of color (SOC) if the demographic data in the results or methods sections included any of the following: race, ethnicity, skin of color, Fitzpatrick scale, sunburn tendency, phototype, skin type, or skin tone.

**Results:**

Out of 76 studies initially identified, only 49 articles met inclusion criteria. Of these 49 articles, only 24 articles recorded data of skin color from their demographics (49%). Subgroup analysis showed no statistically significant difference in the rate of reporting between studies grouped by decade (*p* = 0.779) or by study location (*p* = 0.763).

**Conclusion:**

Darker skin tones can disguise melanotic skin lesions. Less than 50% of RCTs related to melanoma in the top ten international dermatology journals included skin color within their results section to characterize study participants. This has a negative impact on our understanding of this potentially devastating disease.

## Introduction

Skin cancer is the most common malignancy in the United States [1, 2]. While cutaneous malignant melanoma (CMM) accounts for only 1% of all cutaneous malignancies, it has an outsized mortality rate and is responsible for over 80% of skin-cancer deaths [1, 3]. The incidence of CMM is increasing within the United States and has risen more than 325% since 1975 [3]. 

CMM is a malignancy of the melanocytes within the basal layer of the epidermis and encompasses four main recognized subtypes: superficial spreading melanoma (SSM), acral lentiginous melanoma (ALM), nodular melanoma (NM), and lentigo maligna melanoma (LMM). While usually grouped together these diseases are distinct in their pathology, presentation, and epidemiology.

Most CMM affects white males over age 65, which is evidenced by the highly studied incidence in this population [2–4]. Despite the increased relative incidence of CMM in Caucasians, patients with skin of color (SOC), the group most affected by ALM, suffer increased morbidity and mortality when diagnosed with CMM[5]. An increasing body of literature suggests that this may be in part because patients with SOC tend to present with late-stage disease with an increased likelihood of regional and distant metastases [6–9]. It is the purpose of this study to determine if randomized controlled trials (RCTs) involving melanoma in the top ten most impactfcul dermatology journals globally routinely report skin color 

## Methods

### Study design

**Fig. 1:**
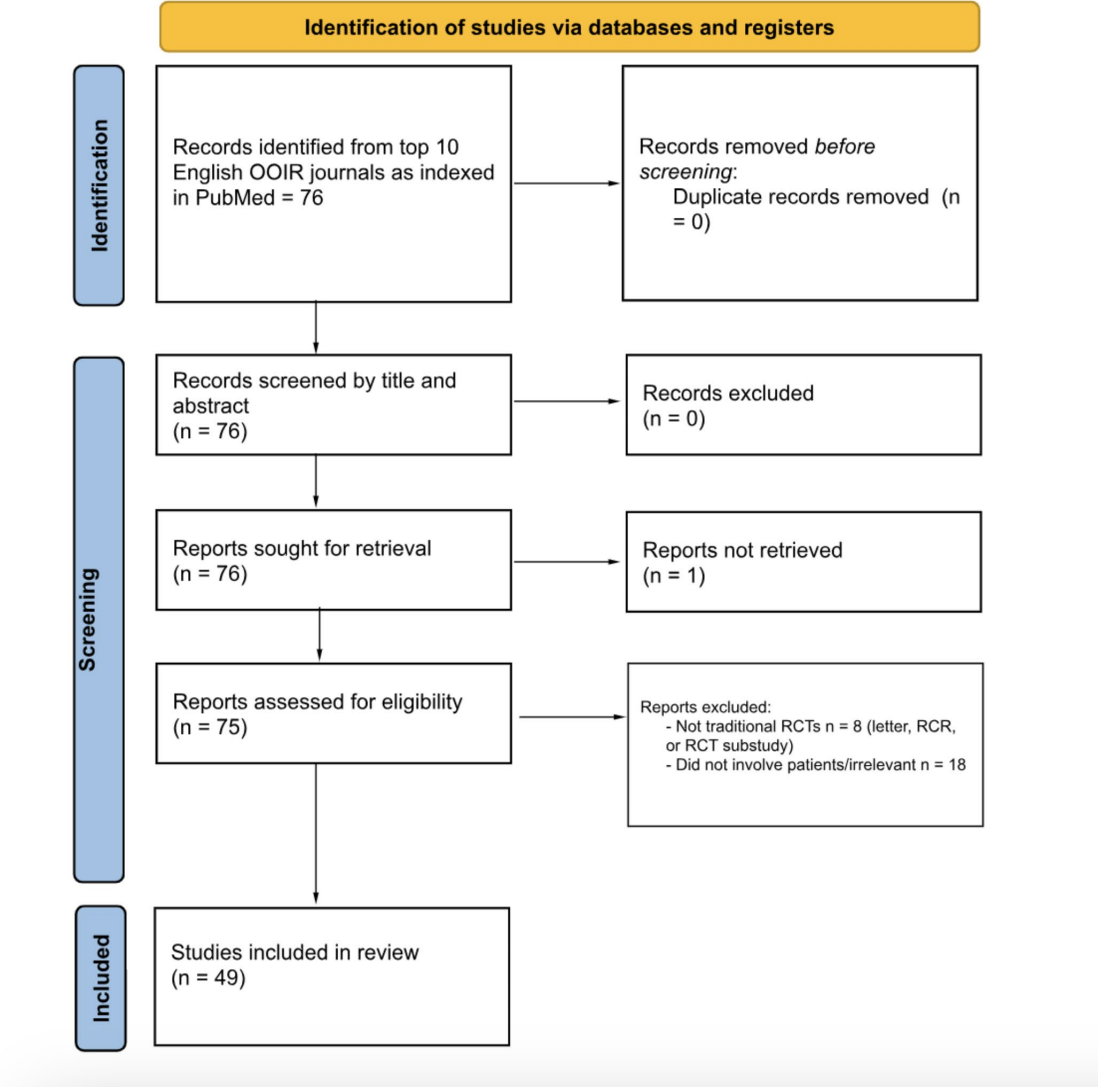
Preferred Reporting Items for Systematic Review and Meta-Analyses (PRISMA) diagram

A systematic review of RCTs involving melanoma was conducted from inception to July 10th, 2023. The ten most impactful dermatology journals globally were included, although they had to be written in English. The journals were selected from the Observatory of International Research rank list (see Table 1.) The 9th ranked journal, Journal der Deutschen Dermatologischen Gesellschaft, was written in Germans, and thus, excluded based on inclusion criteria. The 11th journal, Dermatitis, was chosen to fulfill the top ten written in English criteria. The database PubMed was used for the article search. Search terms included “melanoma” and the abbreviations of the ten journals, which can be seen in Table 1. The filter RCT was applied, but authors had to ensure RCTs were selected while avoiding article types RCT-adjacent-studiessuch as letters to the editor. This study follows the Preferred Reporting Items for Systematic Review and Meta-Analyses (PRISMA), which can be found in Fig. 1.

**Table 1:** List of top ten most impactful dermatology journals globally, from the Observatory of International Research, July 2023

Journal Name:	Impact Factor:	PubMed Term:
Journal of the American Academy of Dermatology	15.49	J Am Acad Dermatol
JAMA Dermatology	11.82	JAMA Dermatology
British Journal of Dermatology	11.11	Br J Dermatol
Journal of the European Academy of Dermatology and Venerology	9.23	J Eur Acad Dermatol Venereol
Journal of Investigative Dermatology	7.59	J Invest Dermatol
Contact Dermatitis	6.42	Contact Dermatitis
American Journal of Clinical Dermatology	6.23	Am J Clin Dermatol
Journal of Dermatological Science	5.41	J Dermatol Sci
Dermatology	5.2	Dermatology
Dermatitis	5.19	Dermatitis

### Inclusion and exclusion criteria

Inclusion criteria included: RCT articles that related to melanoma identification, prevention, or treatment, involved human patients, and were written in English. Exclusion criteria included: non-RCTs, did not involve patients in the study (as seen in RCTs measuring educational initiatives), or if the full text was unavailable.

### Study definitions

For the purpose of this study, the term “melanoma:” is used to refer to any of the four primary subtypes of CMM: acral lentiginous, lentigo maligna, superficial spreading or nodular.

### Article sorting process

All initially collected articles were exported into a research software manager, rayyan.ai, for organization and reference. Articles were screened and sorted, and duplicates were removed by author DC if any existed.

### Data extraction

All included articles were recorded in an analysis spreadsheet where data were extracted by three researchers (DC, NS, JB) into the following categories for each article:

- Journal
- Title
- Author
- Year
- Full Text Available
- Includes skin color/race/ethnicity
- Terms used to describe skin
- Was specific type of melanoma reported? If yes, what type?
- Primary study location

### Statistical analysis

The Statistical Package for the Social Sciences (SPSS) version 29.0 (Armonk, NY; IBM Corp) was utilized for the statistical analysis of this study. Frequency counts and descriptive statistics were used to help report the data. Chi-square testing was used to compare groups due to the nominal variables used in this study. Statistical significance was set at 0.05 for all variables.

### Subgroup analysis

Subgroup analyses were performed on the following categories: publication year grouped by decade (prior to 1999, 2000–2009, 2010–2019, and 2020 and after) and study location (within the United States versus outside the Unites States).

## Results

### Initial search results

A total of 76 articles were retrieved from the initial PubMed search with one article being excluded because full text was not available. Thus, 75 articles were evaluated. 18 of the articles were excluded for not involving patients or were irrelevant to the diagnosis and treatment of melanoma. Eight articles that were not traditional RCTs (RCT-adjacent studies or similar) were also excluded. After article sorting, 49 RCTs were included in this systematic review (see Fig. 1 below).

### Reporting skin color

Of the 49 included articles, only 24 reported skin color (49%). Of the 24 RCTs that reported the race/skin color of participants, 11 of 24 articles (45.8%) utilized either Fitzpatrick scale or sunburn tendency, 5/24 articles (20.8%) utilized race or ethnicity, and 8/24 (33.3%) articles used their own scales.

### Reporting rate by decade

Overall, 24 RCTs reported skin color (49.0%) whereas 25 RCTs did not report skin color (51.0%). For subgroup analysis, prior to 1999 had a 33.3% reporting rate (2/6), 2000–2009 had a 58.3% reporting rate (7/12), 2010–2019 had a 50% reporting rate (11/22), and 2020 to present had a 44.4% reporting rate (4/9) without any statistically significant difference between groups (*p* = 0.779).

### Rate of reporting by study location

A total of 17 RCTs (35.4%) out of 48 articles with identifiable study location (97.9% reported with one article not reporting) were from the United States with the remaining articles (*n* = 31, 64.6%) being conducted outside of the United States. There was no significant difference in the rate of race/skin color reporting based on location within or without the United States (47.1% vs. 51.6%, *p* = 0.763).

## Discussion

In this systematic review of RCTs focused on the diagnosis and treatment of melanoma, skin color was reported less than half the time (49%). This deficiency could compromise our understanding of melanoma and impact the care of patients with SOC. Similar findings were noted in reviews of basal cell carcinoma and squamous cell carcinoma in SOC [10, 11]. 

There was no significant difference in skin color reporting over the past four decades. However, it appears that skin color reporting in RCTs related to melanoma has decreased over the years. Between 2000 and 2009, skin color was reported 58.3% of the time, yet decreased to 50% over the subsequent decade (2010–2019) and has decreased even further to 44.4% after 2020. While statistically insignificant, this decrease highlights a persistent gap in research populations that may be unrepresentative of all skin color types. There was no statistically significant difference between reporting skin color within the United States (47.1%) versus outside the United States (51.6%).

### Education on SOC in dermatology

While only the basics of dermatology are taught to medical students in their preclinical curriculum, there is a disparity in representing SOC. This is a critical deficiency since cutaneous pathologies can appear different on various skin colors, and may impact future patient care [12]. Studies have shown that medical students are not able to diagnose dermatological conditions in darker skin tones as well as in lighter skin tones [13, 14]. While the gap in medical education about dermatological manifestations in patients with SOC is correctable, it will take effort which must, in part, be related to research focusing on patients with SOC.

### Lack of SOC in higher ranked journals

It has been shown time and again that diversity within the dermatologic literature is lacking. One study examined how often SOC content is published in dermatologic journals and found that higher ranked journals published less articles on SOC than lower ranked journals [15]. This highlights the lack of inclusion still seen in medicine today. Conducting studies and publishing peer reviewed articles on patients with SOC needs to be recognized as essential components of medicine and research. Physicians tend to read higher ranked journals to gather new information and see what current studies are being conducted, and if these journals are not publishing articles on SOC, many physicians will never know what has been done in regards to treating or diagnosing patients with SOC.

## Conclusion

Skin color may influence detection, surveillance, treatment, and epidemiology of melanoma. This review determined the rate at which skin color is reported in melanoma-related RCTs in the highest impacting global dermatology journals. More than half of the RCTs fail to report the skin color of the study participants, and this trend does not appear to be improving with time. While melanoma is not the most common skin cancer, it is associated with increased mortality and has increasing in prevalence over the years. Patients with SOC are disproportionately affected by melanoma, which may be due to late-stage diagnosis; thus, including SOC in research is crucial.

## Data Availability

No datasets were generated or analysed during the current study.
